# Morphometric dataset of the alluvial fans at the southern part of Nayband fault, Iran

**DOI:** 10.1016/j.dib.2018.11.017

**Published:** 2018-11-07

**Authors:** Farzaneh Hashemi, Reza Derakhshani, Shahram Shafiei Bafti, Amir Raoof

**Affiliations:** a Department of Geology, Shahid Bahonar University of Kerman, Iran; b Department of Earth Science, Utrecht University, Utrecht, The Netherlands

**Keywords:** Tectonics, Geomorphology, Morphometrics, Geology, Kerman

## Abstract

This data article provides a precise level data on alluvial fans of the western border of Lut desert, a unique location on the Earth, known as one of the hottest spot on the Earth. This data is essential for morphotectonic investigations and is valuable in the field of tectonic and geomorphology studies. It helps to evaluate the region from the viewpoint of tectonic activity by considering the dynamics of alluvial fans, climate and tectonic setting. The data which are presented for 68 quaternary alluvial fans, is taken by processing of remotely sensed Landsat satellite data, photogeology of aerial photographs, measuring on topographic maps and controlled by field checking. This data is useful for defining of a morphotectonic model of this limited access region considering the uplift of the source area along Nayband fault, as the basin–margin fault, respect to the base level.

**Specifications table**

**Table:** 

Subject area	*Geology*
More specific subject area	*Tectonics, Morphotectonics, Tectonic Geomorphology*
Type of data	*Table*
How data was acquired	*Survey, Topographic maps, Photogeology, Field checking*
Data format	*Raw, analyzed*
Experimental factors	*The geometry of alluvial fans*
Experimental features	*The geometric characteristics of Alluvial fans were measured.*
Data source location	*Shahdad, Iran.*
	*Latitude: 57°,33’ to 57°,42’ N & Longitude: 30°,24’ to 30°,55’ E*
Data accessibility	*Data is available with this article.*
Related research article	*N/A*

**Value of the data**

• The data presents a morphotectonic outlook about the activity of Nayband Fault.
• It helps to explain the impact of Nayband fault on the tectonic activity of the Western Lut.
• Data can be applied for quantitative analysis in the field of tectonic geomorphology and morphotectonics.
• Other researchers may use the data for their research work and further investigation.

## 1 Data

The data presented here describe the morphometric characteristic of 68 alluvial fans of western Lut desert. Data is given in table form. The data is prepared based on fieldwork in a limited access region and laboratory analysis.

## 2 Experimental design, materials and methods

Tectonic setting and climatic fluctuations could fundamentally control morphometrics of alluvial fans, the prominent geomorphological landforms in mountain fronts, by uplifting and maintaining the topography, controlling the sediment supply and duration of deposition through increasing gradients of drainages [1], [2], [3], [4], [5], [6], [7], [8], [9], [10] especially in arid zones where little changes in precipitation can effectively affect stream discharge [11]. These landforms that could be served as important groundwater reservoirs [12], [13] made out of loose rock materials that are formed at the mountain fronts where drainages reach the plain. In a mountain range, some of the alluvial fans show different characteristics which are important as they present the history of the changes that have taken place in the region over millennia. They give evidence of tectonic activities and the past climate that had formed the morphology of these landforms and the challenge is to understand the relative impact of each.

In order to achieve this goal, each of the alluvial fans was first mapped through satellite studies and photogeology. Subsequently, their position and boundary were controlled and finalized in the field. Thus, some characteristics such as the environment, area, length and width of alluvial fans were calculated by using the mentioned checked map. To calculate other parameters, such as minimum elevation, average elevation, maximum elevation, elevation gain, elevation loss, maximum slope and the average slope of alluvial fans, after determination of the location of the points on a topographic map, the elevation of them was controlled by GPS on the field.

Iran is considered to be tectonically active regarding its geological situation and features [14], [15], [16], [17], [18], [19] and this paper has tried to present data valuable for examining the morphotectonic imprints which are discernible in the morphology of the fans in the southern part of Nayband fault, a right lateral strike slip fault in Central Iran, that forms the western border of Lut desert. Due to the close distance of these landforms to one of the area wherein five of the seven years was detected as the hottest spot on the Earth, having a surface temperature above 70 °C [20], access to them is not easy and the present data is also valuable at this point of view.

**Fig. 1:**
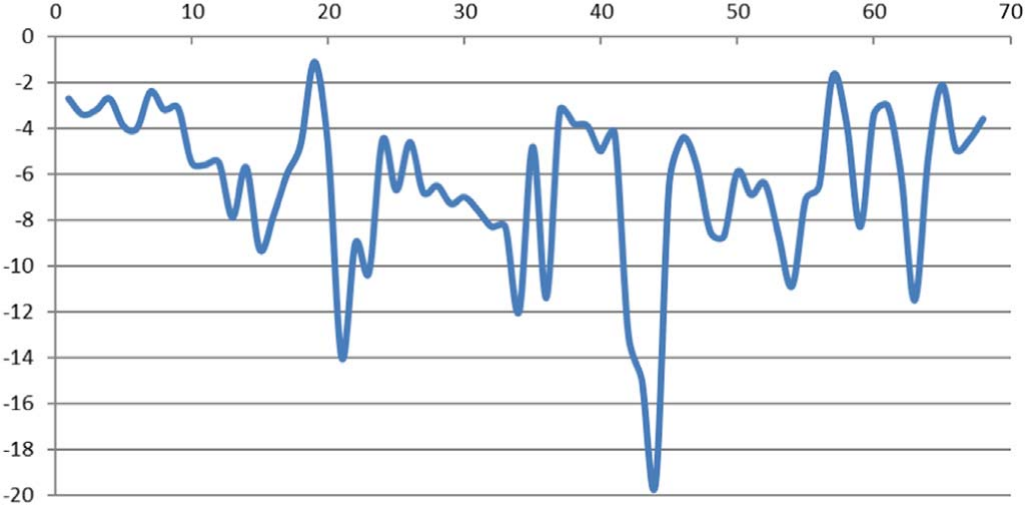
Alluvial fans average slope. Horizontal axis shows the number of the alluvial fan and vertical axis shows the measured average slope in percentage for each of the alluvial fans.

**Fig. 2:**
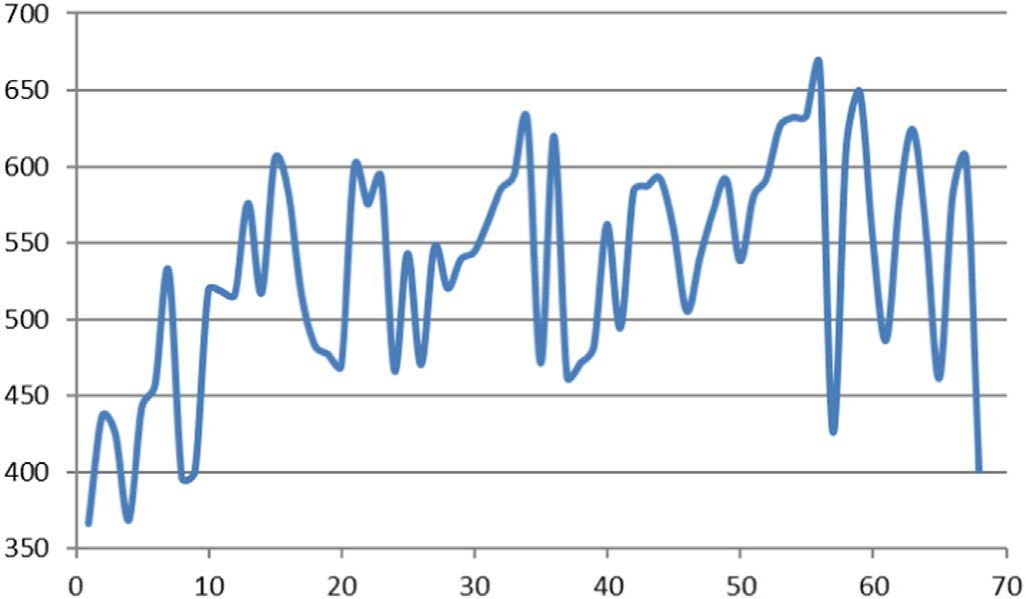
Average elevation of Alluvial fans in western border of Lut desert. Horizontal axis shows the number of the alluvial fan and vertical axis shows the average elevation in meter of each of the alluvial fans from the sea level.

**Fig. 3:**
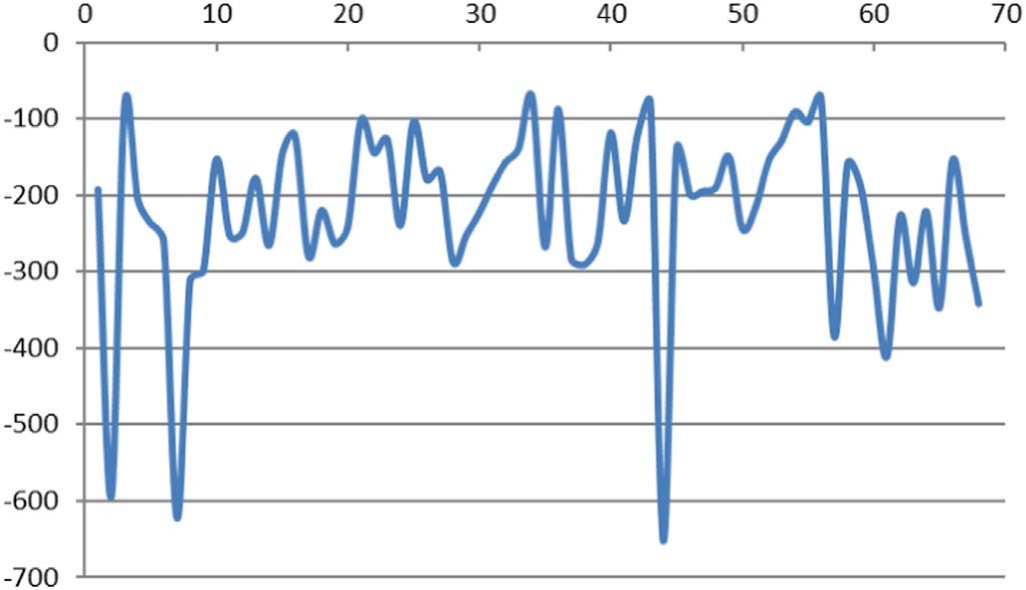
Alluvial fans elevation loss. The number of alluvial fans is shown in the horizontal axis while the vertical axis shows the elevation loss in meter for each of the alluvial fans.

**Fig. 4:**
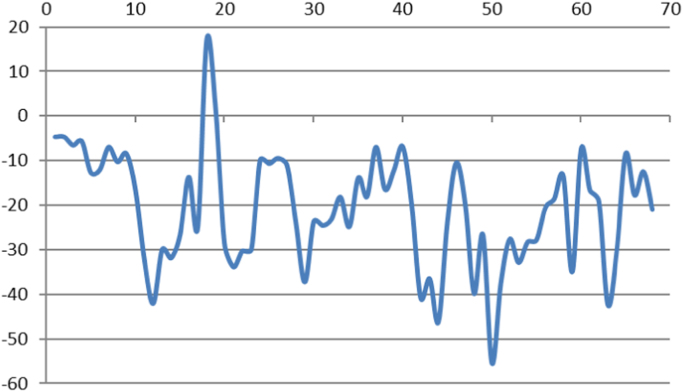
Maximum slope of alluvial fans. The number of alluvial fans is shown in the horizontal axis while the vertical axis shows the maximum slope in percent for each of the alluvial fans.

**Fig. 5:**
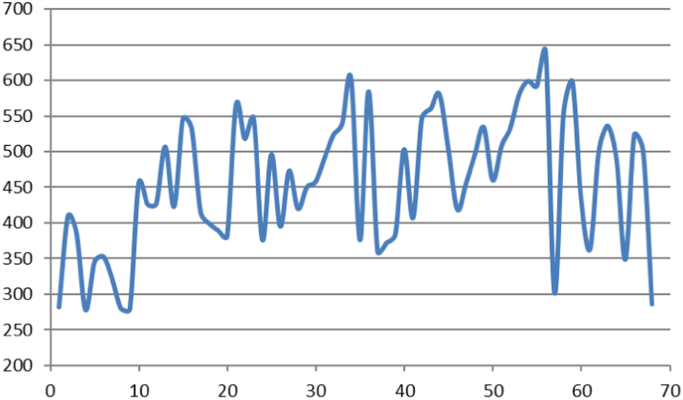
Minimum elevation of alluvial fans in the study area. The number of alluvial fans is shown in the horizontal axis while the vertical axis shows the minimum elevation in meter from the sea level for each of the alluvial fans.

The morphometric attributes of alluvial fans which are presented here (Table 1, Table 2 and Fig. 1, Fig. 2, Fig. 3, Fig. 4, Fig. 5) could help to understand the tectonic activity stage that has governed the morphology of the features. Thus, the present data is valuable to analyse the morphotectonic control over the alluvial fan morphology by doing a comparative analysis of these fans with respect to morphological attributes.

**Table 1:** Geometric data of alluvial fans. N: Alluvial fan number, L: Length of the Alluvial fan in meters, W: Width of the Alluvial fan in meters, P: Perimeter of the Alluvial fan in meters, A: Area of the Alluvial fan in square meters.

N	L	W	P	A
1	7350	1270	16.173	6152318
2	1770	331	3869	393789
3	2530	354	5506	617922
4	7440	2130	18052	13106070
5	6150	866	13520	4226489
6	6610	769	20889	9822912
7	2660	867	20982	7624863
8	9840	714	20932	7450879
9	9720	177	6017	543856
10	2760	1320	14010	6066108
11	4560	1570	10827	5067842
12	4530	758	9925	2864439
13	2240	237	5055	473821
14	4640	691	10299	2601741
15	1580	244	3366	265993
16	1590	148	3407	161264
17	4690	741	10840	3637627
18	4700	693	10942	3378624
19	4950	553	10584	2265571
20	5240	295	11058	1213185
21	7240	147	1809	79526
22	1610	357	3548	391794
23	1230	354	3282	456287
24	5380	789	11469	2475554
25	1540	168	3403	179170
26	3930	205	8838	1074196
27	2500	315	6169	515950
28	4110	872	10389	3517202
29	343	414	7646	1290645
30	3190	636	7385	1448130
31	2460	244	6470	731873
32	1890	488	4617	756267
33	1670	160	4270	311460
34	594	146	1688	83169
35	5580	422	12044	1878376
36	766	160	2189	137841
37	8270	5800	24279	36799188
38	7620	476	16203	3781584
39	6710	1670	16233	8145812
40	2400	985	7047	1910312
41	5650	886	13948	3961240
42	934	157	2378	151441
43	526	217	1776	98149
44	370	178	1374	54221
45	2210	776	6327	1533558
46	4540	772	10181	2466201
47	3490	1300	9425	3807583
48	2230	659	5658	1186220
49	1720	647	4723	936146
50	4120	1500	12898	5193159
51	3090	926	7661	2156896
52	2440	1500	7042	2927413
53	1490	491	3775	663634
54	831	645	2906	431537
55	1460	1550	6746	2090894
56	1100	902	4059	789848
57	2420	22600	92718	486826249
58	4210	976	10022	2992619
59	2260	1280	5685	1955055
60	8820	1770	20821	13401104
61	1440	3030	32971	36204138
62	3740	867	9832	2738026
63	2700	745	6501	1245319
64	4340	1140	9396	2927855
65	1690	8760	45684	128145633
66	3120	1240	7910	3175471
67	5730	1190	13498	4872803
68	956	7130	66538	172077148

**Table 2:** Alluvial fans elevation data. N: Alluvial fan number, Em: Minimum elevation of alluvial fan in meters, Ev: Average elevation of alluvial fan in meters, Ex: Maximum elevation of alluvial fan in meters, Eg: Gained elevation of alluvial fan in meters, Es: Lost elevation of alluvial fan in meters, Sx: Maximum slope of alluvial fan in percent, Sv: Average slope of alluvial fan in percent.

N	Em	Ev	Ex	Eg	Es	Sx	Sv
1	282	366	475	0.07	−193	−4.6	−2.7
2	409	436	468	0	−594	−4.6	−3.4
3	385	425	468	0.79	−83	−6.5	−3.2
4	278	368	480	0.58	−203	−5.6	−2.7
5	345	442	582	0	−237	−12.6	−3.9
6	353	457	614	0.04	−261	−12	−4
7	322	532	941	2.97	−622	−6.9	−2.4
8	280	396	592	0	−312	−10.2	−3.2
9	279	400	579	0.88	−300	−8.4	−3.1
10	456	519	608	0	−153	−16.5	−5.5
11	426	518	680	0	−253	−32.2	−5.6
12	427	516	677	0	−249	−42	−5.5
13	507	576	685	0	−178	−29.9	−7.9
14	423	517	688	0.21	−266	−31.8	−5.7
15	546	605	692	0	−146	−26.6	−9.3
16	532	584	655	0	−123	−13.6	−7.8
17	415	517	696	0	−280	−24.9	−6
18	399	483	618	0.32	−220	17.2	4.7
19	389	477	652	0.57	−264	1.4	1.1
20	381	469	623	0	−242	−28.5	−4.8
21	565	600	667	0	−102	−33.8	−14
22	518	575	664	0	−145	−30.2	−9
23	546	593	674	0	−128	−29.9	−10.3
24	376	466	616	0	−240	−9.8	−4.5
25	496	543	600	0	−104	−10.6	−6.7
26	395	470	575	0	−180	−9.4	−4.6
27	473	547	642	0	−169	−11	−6.8
28	420	520	687	0	−287	−23.7	−6.5
29	450	539	702	0	−253	−37.2	−7.3
30	458	544	681	0	−223	−23.6	−7
31	491	563	678	0	−187	−24.5	−7.6
32	523	585	680	0	−157	−23.1	−8.3
33	539	594	678	0	−139	−18.1	−8.3
34	602	630	673	0	−71.3	−24.8	−12
35	376	471	646	0	−268	−13.8	−4.8
36	584	620	671	0.05	−87.5	−18	−11.4
37	360	462	622	0.15	−283	−6.9	−3.2
38	372	471	663	0.42	−291	−16.4	−3.8
39	384	482	647	0	−263	−12.1	−3.9
40	503	562	622	0	−119	−6.8	−5
41	407	494	639	1.81	−234	−19.8	−4.2
42	547	584	671	0	−124	−40.7	−13
43	560	587	639	0	−79.6	−36.4	−15
44	580	592	644	2.3	−652	−46.2	−19.5
45	503	559	644	0	−141	−23.9	−6.4
46	419	505	618	0	−199	−10.4	−4.4
47	456	541	651	0	−195	−19.8	−5.6
48	496	571	686	0.07	−190	−39.8	−8.5
49	534	591	684	0.02	−150	−26.6	−8.7
50	460	538	704	0.36	−244	−55.4	−5.9
51	508	580	725	0	−216	−37.3	−6.9
52	533	592	690	0	−156	−27.5	−6.4
53	580	626	709	0	−129	−32.8	−8.6
54	599	632	690	0	−91	−28.2	−10.9
55	592	633	697	0	−105	−27.7	−7.1
56	639	666	712	0	−72.9	−20.5	−6.5
57	302	426	686	3.33	−385	−18.5	−1.7
58	552	613	712	0.2	−160	−13.4	−3.8
59	598	649	787	0	−189	−34.9	−8.3
60	433	552	731	0	−297	−7.3	−3.4
61	363	486	773	1.87	−411	−16.6	−3
62	501	578	728	0	−227	−19.1	−6.1
63	536	624	851	0	−315	−42.3	−11.5
64	489	558	709	0.7	−221	−30.2	−5.2
65	349	461	695	1.44	−347	−8.4	−2.1
66	523	581	678	0	−154	−17.7	−4.9
67	501	605	756	0.16	−255	−12.4	−4.5
68	286	401	628	0	−342	−20.9	−3.6
