# Supplementary material for: Morphometric dataset of the alluvial fans at the southern part of Nayband fault, Iran
Source: Data Brief. 2018 Nov 7;21:1756–63. doi: 10.1016/j.dib.2018.11.017 (PMC6249545; doi:10.1016/j.dib.2018.11.017)
Supplement: Supplementary file 1 — Supplementary material [file mmc1.docx]

Conflict of Interest Form

The authors have no conflict of interest regarding this article.

List of authors:

Farzaneh Hashemi, Reza Derakhshani, Shahram Shafiei Bafti, Amir Raoof
